# A genotyping method combining primer competition PCR with HRM analysis to identify point mutations in Duchenne animal models

**DOI:** 10.1038/s41598-020-74173-y

**Published:** 2020-10-14

**Authors:** Haizpea Lasa-Fernandez, Laura Mosqueira-Martín, Ainhoa Alzualde, Jaione Lasa-Elgarresta, Ainara Vallejo-Illarramendi

**Affiliations:** 1 Group of Neurosciences, Dept. Pediatrics, University of the Basque Country UPV/EHU, Hospital Universitario Donostia, Paseo Dr. Begiristain 105, 20014 San Sebastián, Spain; 2 Group of Neuromuscular Diseases, Biodonostia Health Research Institute, Paseo Dr. Begiristain s/n, 20014 San Sebastián, Spain; 3 CIBERNED, Instituto de Salud Carlos III, 28031 Madrid, Spain; 4 BIOBIDE, San Sebastián, Spain

**Keywords:** Genotyping and haplotyping, PCR-based techniques, Neuromuscular disease

## Abstract

Dystrophin-null *sapje* zebrafish is an excellent model for better understanding the pathological mechanisms underlying Duchenne muscular dystrophy, and it has recently arisen as a powerful tool for high-throughput screening of therapeutic candidates for this disease. While dystrophic phenotype in *sapje* larvae can be easily detected by birefringence, zebrafish genotyping is necessary for drug screening experiments, where the potential rescue of larvae phenotype is the primary outcome. Genotyping is also desirable during colony husbandry since heterozygous progenitors need to be selected. Currently, *sapje* zebrafish are genotyped through techniques involving sequencing or multi-step PCR, which are often costly, tedious, or require special equipment. Here we report a simple, precise, cost-effective, and versatile PCR genotyping method based on primer competition. Genotypes can be resolved by standard agarose gel electrophoresis and high-resolution melt assay, the latter being especially useful for genotyping a large number of samples. Our approach has shown high sensitivity, specificity, and reproducibility in detecting the A/T point mutation in *sapje* zebrafish and the C/T mutation in the mdx mouse model of Duchenne. Hence, this method can be applied to other single nucleotide substitutions and may be further optimized to detect small insertions and deletions. Given its robust performance with crude DNA extracts, our strategy may be particularly well-suited for detecting single nucleotide variants in poor-quality samples such as ancient DNA or DNA from formalin-fixed, paraffin-embedded material.

## Introduction

Duchenne muscular dystrophy (DMD) is the most common lethal childhood neuromuscular disease, and it is caused by loss of function mutations in the X-linked *DMD* gene that codes for dystrophin protein. Boys with DMD undergo progressive weakness and muscle degeneration that result in loss of ambulation around the age of 12, and premature death in their 20s or early 30s^1^.

Dystrophin-null mice (mdx) have been widely used to study the pathogenesis of DMD, and the efficacy of novel therapies. However, compared to the severe clinical manifestations observed in Duchenne boys, mdx mice present a considerably milder phenotype, with only a minor reduction in lifespan^2^. The zebrafish DMD model sapta222a, namely *sapje*, carries a point mutation in the dystrophin orthologous gene^3^. S*apje* zebrafish present severe muscle disorganization, progressive motor dysfunction and early death by 10–12 days post fertilization (dpf)^4^, and thus, compared to the mdx mice, *sapje* zebrafish recapitulate to a greater extent the human disease. This muscle disorganization can be easily detected by birefringence under polarized light in zebrafish larvae at 3–4 dpf^5^. Also, the early lethality of *sapje* at larvae stages greatly expedites survival experiments. These features make the *sapje* zebrafish a powerful model system for whole organism high throughput screening. Additional advantages of *sapje* zebrafish over other DMD animal models are their smaller size and transparency at larvae stages, a large number of offspring every one or two weeks, and lower maintenance costs^6^. These advantages have likely contributed to an increased use of this model, particularly in preclinical studies focused on testing the efficacy of therapeutic candidates for Duchenne^4,7–9^.

Since *sapje* mutants die at the larvae stage, heterozygous (Het) progenitors are needed, and therefore proper genotyping is useful in order to establish *sapje* colonies and subsequent crosses. Moreover, in drug screening assays in which the aim is to rescue the dystrophic phenotype, it is often mandatory to genotype the larvae^4^. The most common methods to genotype *sapje* mutants are Sanger sequencing, and the derived cleaved amplified polymorphic sequences method (dCAPS)^10^, a technique that introduces or destroys restriction enzyme recognition sites by using primers containing one or more mismatches to the template DNA. The modified PCR product is then subjected to restriction enzyme digestion and the presence or absence of single-nucleotide mutations is determined by the resulting restriction pattern. However, these techniques are expensive, tedious, and/or require special equipment. Thereby, a simple genotyping method would considerably facilitate the use of *sapje* zebrafish in preclinical studies in laboratories and biotech companies worldwide. In this study, we aimed to develop a simple, reliable, and cost-effective genotyping protocol that would facilitate studies in *sapje* zebrafish.

## Results

### Standard HRM assay

First, we tested a fast DNA extraction method based on an alkaline lysis protocol^11^. Using this method, we have been able to extract DNA from fresh and paraformaldehyde-fixed zebrafish samples (larvae and adults) with enough yield and quality for PCR amplification. Next, we aimed to use the High-Resolution Melt (HRM) assay for genotyping *sapje* zebrafish. HRM analysis is a post-PCR analysis method used to identify variations in nucleic acid sequences. This method is based on detecting small differences in PCR dissociation curves by measuring the change of fluorescence intensity in the transition from double-stranded to single-stranded DNA. This technique is highly convenient when analysing a large number of samples since it does not rely on electrophoresis for analysis. It is commonly used to discriminate single point mutations in PCR products up to 300 bp^12^. In fact, it has been previously used to genotype mdx mice carrying a C-to-T point mutation^13^, and therefore, we aimed to apply this method for genotyping *sapje* zebrafish.

**Figure 1:**
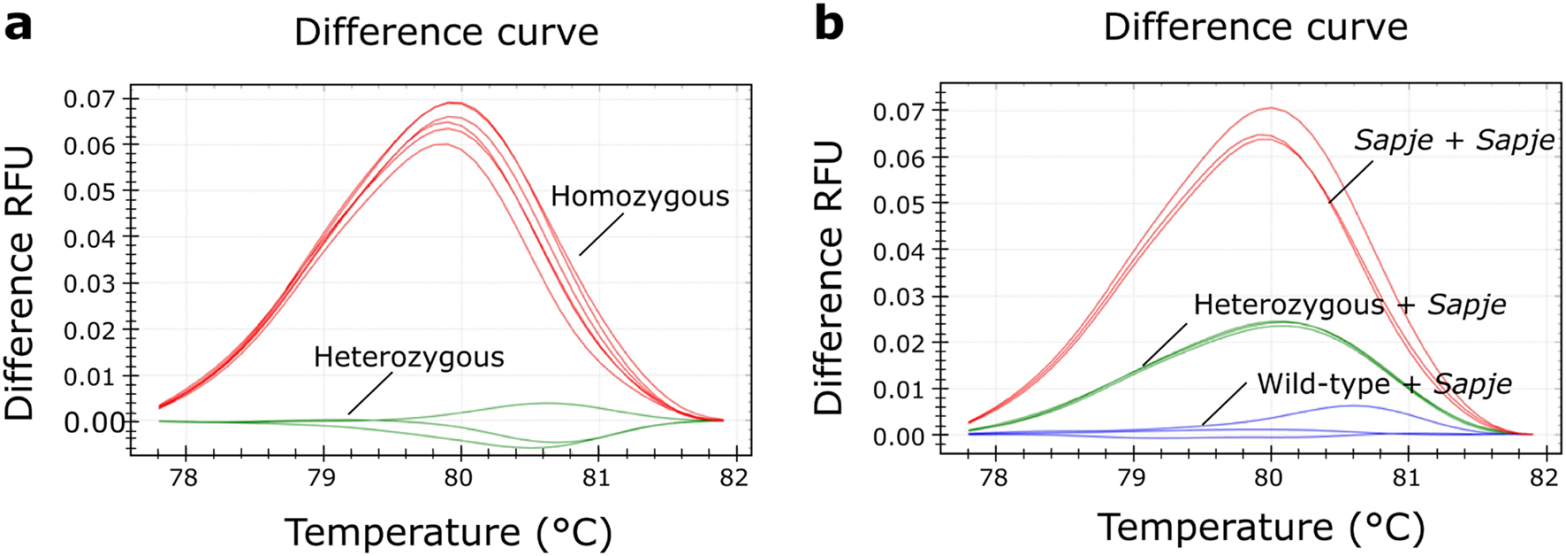
Representative high resolution melting graphs from standard PCR-HRM assay. (**a**) HRM difference melt plot of three zebrafish samples with known genotypes: a wild-type, a *sapje,* and a heterozygous sample. PCR-HRM analysis was performed in triplicate with the heterozygous cluster as a baseline. The samples were assigned to two different clusters: homozygous (wild-type and *sapje*, red) and heterozygous (green). (**b**) HRM difference melt plot of three DNA samples from zebrafish larvae diluted 1:1 with a *sapje* sample. This analysis was performed in triplicate, with the wild-type as a baseline. The samples were assigned to three different clusters corresponding to *sapje* (red), heterozygous (green), and wild-type (blue) genotypes.

To maximize the melting difference between wild-type (WT) and *sapje* samples, we designed a primer pair that amplifies a short PCR product of 166 bp (standard HRM primer pair, Table 1). Subsequently, we performed an HRM analysis on three samples with known genotypes: a WT, a *sapje,* and a Het sample (Fig. 1). This analysis was able to discriminate between the homozygous and Het samples, but it was not able to discriminate between *sapje* and WT homozygous samples (Fig. 1a). This result was not wholly unexpected since the single A/T point mutation present in *sapje* zebrafish is the most challenging base change for HRM detection due to the small melt curve shift (< 0.2 °C). In contrast, mdx mice^13^ carry a C/T point mutation that results in a broader temperature shift (0.8 °C).

**Table 1:** PCR primers and expected PCR products.

Method	Primer name	Primer sequence (5′ → 3′)	Product size (bp)
**Standard HRM**	HRM-For	TTCATTTGCAATGGATGCTCAA	166
	HRM-Rev	AATAGTAAAACAGCCAGCTGAACCA	
**pcPCR**	pcPCR-For	GCGCGTTCATTTGCAATGGATGCTCAA	
	pcPCR-WT-Rev	GATACGCTGCTTTAATGCCTTTAACTCGAGTGAAGCCACGTTCTT**T**	107
	pcPCR-Mut-Rev	CGGCCACTCGAGTGAAGCCACGTTCTT**A**	89

Bold letters represent allele-specific nucleotides at the position of the *sapje* mutation. Non-specific synthetic tails are underlined.

We then used a common strategy to identify the A/T base change by HRM, which is to dilute sample DNA with homozygous DNA^14^. Indeed, when we diluted the three DNA samples with a known *sapje* sample (1:1) and performed HRM analysis, we were able to identify the three different genotypes precisely (Fig. 1b). However, the addition of an extra PCR and dilution step per sample substantially increases the risk of contamination, the hands-on time, and the costs per sample, and thus, we decided to try a more straightforward approach.

### Primer competition PCR

**Figure 2:**
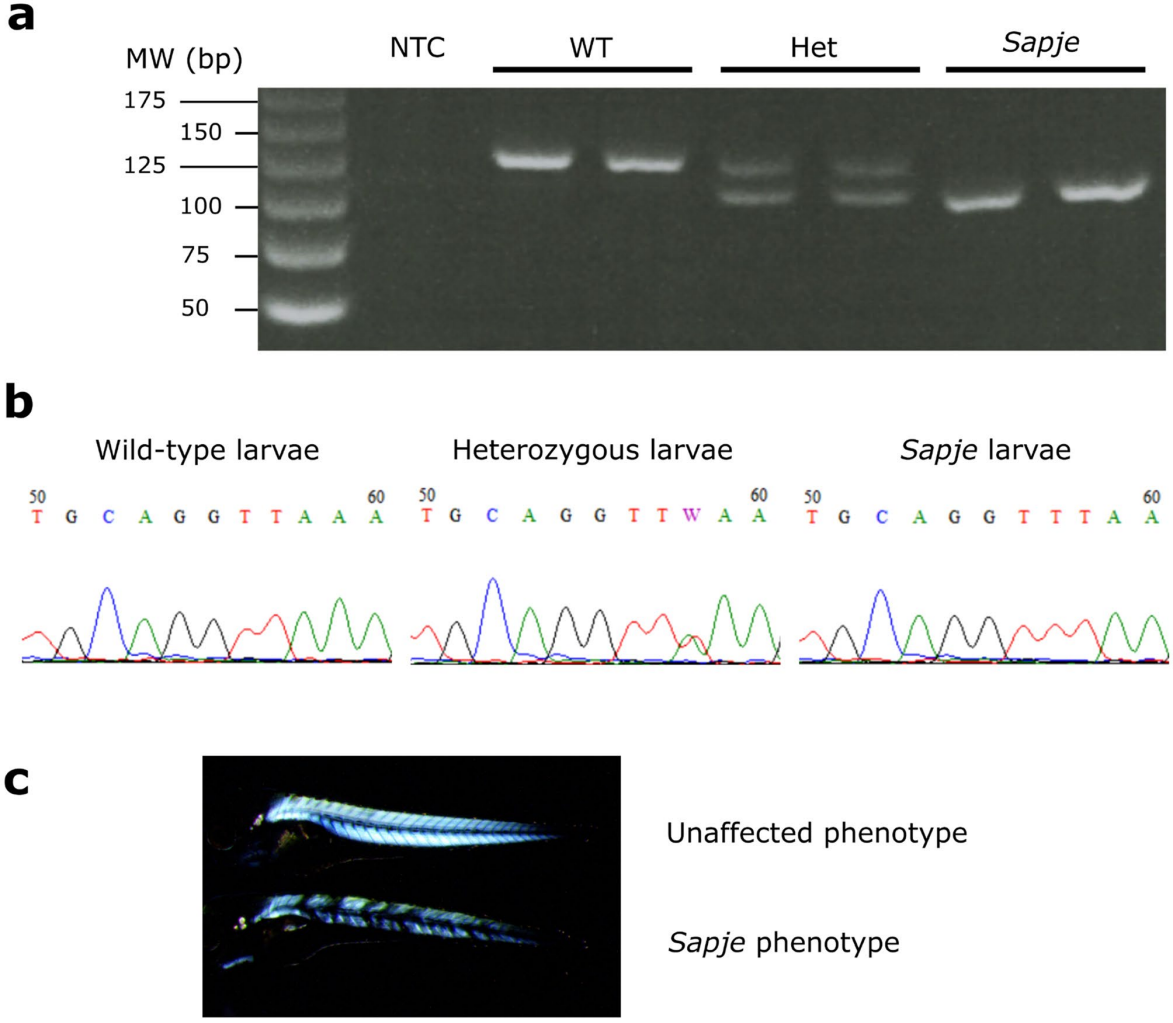
Validation of primer competition PCR for *sapje* zebrafish genotyping. (**a**) Genotypes of two wild-types, two heterozygous and two *sapje* mutants, were resolved by agarose electrophoresis after pcPCR. PCR products corresponded with the expected band sizes. No bands were observed in the non-template control (NTC), and no primer dimers were detected. (**b**) Representative Sanger analysis of wild-type, heterozygous, and *sapje* samples. (**c**) Images illustrate representative unaffected and *sapje* phenotypes of zebrafish larvae by birefringence analysis.

In order to avoid the extra dilution step, we decided to use an approach based on the primer competition PCR technique (pcPCR), in which allele-specific primers compete in a single PCR that result in genotype specific products that differ in length^15^. This technique has been previously optimized for genotyping several strains of DMD mice carrying different point mutations^16^. Hence, we designed a primer trio that produces different sized amplification products for WT and *sapje* alleles, by using the non-specific synthetic tails at the 5′ ends described in Shin et al. ^16^, and allele-specific nucleotides at the 3′ end of the reverse primers (A or T, Table 1). The optimal annealing and extension temperature was elucidated through gradient PCR with temperatures ranging from 60 to 65 °C and subsequent agarose gel electrophoresis. Lower annealing temperatures resulted in the amplification of non-specific bands in homozygous samples (see Supplementary Fig. S1 online). In contrast, at 65 °C, pcPCR products analysed by 3% agarose gel electrophoresis showed the expected pattern for WT, Het, and *sapje* samples with no detection of additional bands (Fig. 2a). Genotypes of several samples were also resolved by Sanger sequencing using HRM-for and HRM-rev primers (Table 1), with concordant results (Fig. 2b, Table 2). Likewise, birefringence analysis showed that zebrafish genotyped as *sapje* specifically presented a disorganized muscle pattern that is characteristic of the dystrophic phenotype, while zebrafish genotyped as WT or Het showed a regular muscle pattern (Fig. 2c).

**Table 2:** Genotype and phenotype analyses in a subset sample of zebrafish larvae.

Sample	Phenotype	Cluster	Confidence %	Tm	HRM	Gel	Seq
1	*Sapje*	2	99.8	79*.*17 ± 0*.*06	*Sapje*	*Sapje*	*Sapje*
6	Unaffected	1	99.4	78*.*8 ± 0*.*0	Het	Het	Het
8	*Sapje*	2	99.3	79*.*13 ± 0*.*06	*Sapje*	*Sapje*	*Sapje*
9	Unaffected	1	94.6	78*.*77 ± 0*.*06	Het	Het	Het
16	*Sapje*	2	97.9	79 ± 0*.*10	*Sapje*	*Sapje*	*Sapje*
21	*Sapje*	2	99.3	79*.*1 ± 0*.*0	*Sapje*	*Sapje*	*Sapje*
22	Unaffected	3	98.4	78*.*70 ± 0*.*0	WT	WT	WT
23	Unaffected	1	99.6	78*.*8 ± 0*.*0	Het	Het	Het
27	Unaffected	3	98.3	78*.*7 ± 0*.*0	WT	WT	WT
28	*Sapje*	2	60.4	78.9	*Sapje*	*Sapje*	*Sapje*
		1	97.7	78.9	Het		
		2	98.0	79	*Sapje*		
39	Unaffected	3	98.2	78*.*7 ± 0*.*0	WT	WT	WT
40	Unaffected	1	71.7	78.6	Het	Het	Het
		1	98.3	78.7	Het		
		3	92.6	78.6	WT		
41	*Sapje*	2	99.3	79*.*1 ± 0*.*0	*Sapje*	*Sapje*	*Sapje*
43	*Sapje*	2	99.6	79 ± 0*.*0	*Sapje*	*Sapje*	*Sapje*
46	Unaffected	1	99.7	78*.*7 ± 0*.*0	Het	Het	Het
54	Unaffected	1	99.7	78*.*73 ± 0*.*06	Het	Het	Het
55	Unaffected	3	99.2	78*.*77 ± 0*.*06	WT	WT	WT
56	Unaffected	3	99.2	78*.*73 ± 0*.*06	WT	WT	WT

*Sapje* phenotype was determined by birefringence analysis. HRM data shows genotypes resolved by HRM analysis of pcPCR products. Cluster, percentage confidence (highest value), and melting temperature (Tm, mean ± SD) values are extracted from the HRM analysis. Gel, refers to genotypes resolved by 3% agarose gel electrophoresis. Seq, refers to genotypes resolved by Sanger sequencing. The entire list of 60 samples can be found as Supplementary Table S1 online.

**Figure 3:**
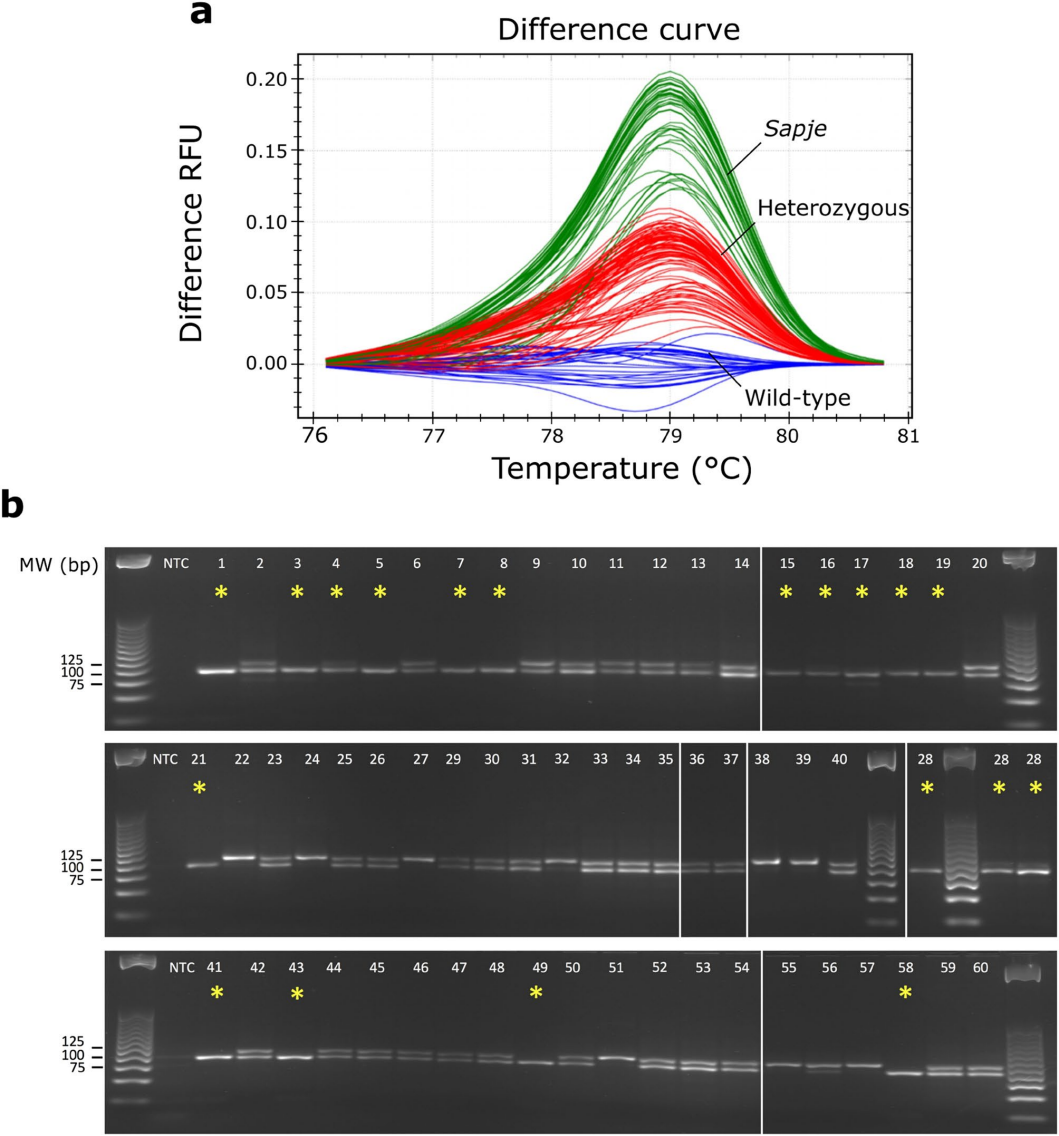
Genotyping *sapje* zebrafish by primer competition PCR followed by HRM and agarose electrophoresis. (**a**) HRM difference melt plots of 60 zebrafish larvae performed in triplicate with the wild-type cluster as a baseline. 58 samples were automatically assigned to three different clusters with 100% reliability, but two samples were inconclusive. Wild-type (blue), heterozygous (red), and *sapje* (green) clusters are identified on the melt plot. (**b**) 100% of the genotypes were resolved after running pcPCR products (same as in **a**) in a 3% agarose gel electrophoresis. Cropped images from two different gels are shown. White lines depict non-consecutive lanes, while yellow asterisks depict *sapje* genotypes.

Next, we aimed to determine the sensitivity and reliability of this method. Additionally, we wanted to establish whether HRM analysis could also be used to resolve zebrafish genotypes after pcPCR amplification since this would substantially reduce the hands-on time required for agarose electrophoresis. To test this, we used DNA from 60 zebrafish larvae, 17 of which presented a dystrophic phenotype (Table 2; Supplementary Table S1 online). These samples underwent pcPCR as described above, and PCR products were analysed in triplicate by HRM assay (Fig. 3a). All samples tested were amplified and resulted in a corresponding melting curve. HRM assay was able to efficiently discriminate WT, Het, and *sapje* samples in an unbiased manner, based on pcPCR product melt curve profiles. After HRM analysis, 97% of samples were automatically assigned to one of the three clusters corresponding to WT (cluster 3), *sapje* (cluster 2), or Het genotypes (cluster 1). Two samples (#28 and #40) were termed as unresolved since their replicates were assigned to two different clusters. The HRM assay was re-run a second time, and this allowed resolution of sample #40 as Het since all the three replicates assigned to this cluster, with 91, 94, and 98% confidence. The genotype of each cluster was determined, by performing Sanger analysis in at least five samples per cluster, and later on by agarose electrophoresis of pcPCR products (Table 2; Supplementary Table S1 online). The reliability of the melt curve analysis in separating each genotype from the other two was 100% in 58 samples, with a 90% confidence threshold reached in at least one replicate per sample. The sensitivity of the melt curve analysis was 98%, due to one false negative in a replicate from sample #28. Sensitivity was calculated as the percentage of *sapje* replicates correctly identified as positive by the assay (50/51). The specificity of the pcPCR-HRM assay was 100%, calculated as the percentage of phenotypically unaffected replicates (Het or WT) correctly identified as negative by the assay (129/129).

We then analysed the 60 PCR products by agarose electrophoresis (Fig. 3b), and these results were concordant with the genotypes previously resolved by HRM analysis. Gel electrophoresis of the pcPCR products from samples #28 and #40 was able to identify them unquestionably as a *sapje* and a Het, respectively. Moreover, these genotypes were concordant with data obtained by Sanger sequencing and birefringence phenotyping. In summary, the resolution of pcPCR products by combining HRM and agarose electrophoresis allowed the genotyping of all the zebrafish samples with 100% accuracy. Indeed, our technique resulted in 100% genotype–phenotype correlation, where the 17 samples identified as *sapje* by the birefringence analysis were genotyped as A/T mutants with the pcPCR method (Fig. 3; Supplementary Table S1 online).

**Figure 4:**
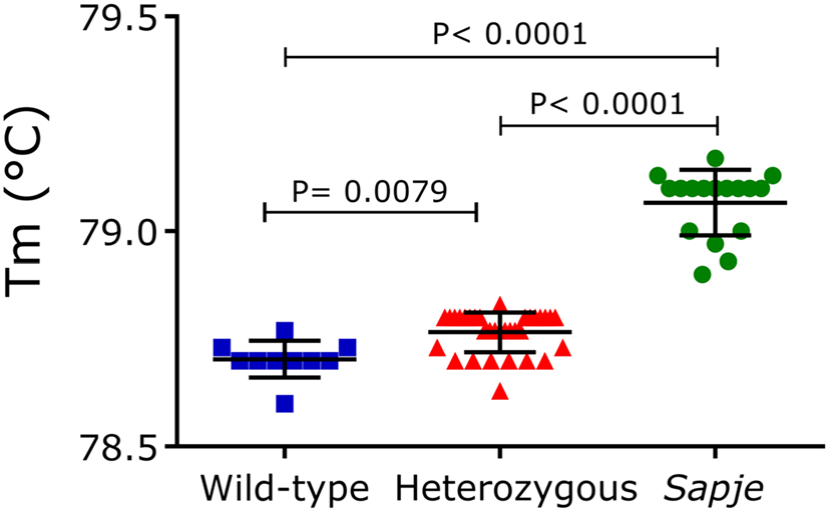
Analysis of melting temperatures (Tm) from pcPCR products. Average Tm values of 60 zebrafish larvae measured in triplicate: wild-type (blue), heterozygous (red), and *sapje* (green) genotypes.. Data are expressed as mean ± SD. One-way ANOVA with Tukey’s multiple comparisons test was used for statistical analysis.

Interestingly, we found that the melting temperature (Tm) of the pcPCR products from *sapje* samples (79.07 ± 0.08) were significantly higher compared to WT (78.70 ± 0.04) and Het samples (78.77 ± 0.05), with an increase in the mean Tm of 0.37 °C and 0.3 °C compared to WT and Het samples, respectively (p < 0.0001; Fig. 4). We also observed minor, although statistically significant differences in the Tm between WT and Het samples (0.07 °C, p = 0.0079). This result is in agreement with the Tm values calculated by Oligo Calc in silico calculator (https://biotools.nubic.northwestern.edu/OligoCalc.html), which is 0.3 °C higher in *sapje* PCR products (75.9 °C in WT vs. 76.2 °C in *sapje*). Overall, our results indicate that Tm analysis is able to discriminate *sapje* samples from healthy unaffected samples (WT or Het), without the need to perform a thorough HRM analysis or agarose electrophoresis. However, differentiation between WT and Het genotypes may be challenging when only Tm values are used for analysis. Genotype-specific Tm values were also used for determining the intra- and inter-assay variability on three runs on different days (Table 3). The intra-assay coefficients of variation (CVs) and inter-assay CVs showed values ranged from 0.04 to 0.13%, and from 0.03 to 0.07%, respectively. These values are well below the maximum acceptance criteria for precision in medicine (< 5–10%).

**Table 3:** Determination of the intra- and inter-assay reproducibility.

	Run 1		Run 2		Run 3		Inter assay	
	Tm	CV%	Tm	CV%	Tm	CV%	Tm	CV%
*Sapje* (n = 17)	79*.*07 ± 0*.*08^∗^	0.10	79*.*10 ± 0*.*06^∗^	0.08	79*.*05 ± 0*.*10^∗^	0.13	79*.*07 ± 0*.*03^∗^	0.03
WT (n = 10)	78*.*70 ± 0*.*04^###^	0.05	78*.*75 ± 0*.*05^##^	0.07	78*.*67 ± 0*.*06	0.07	78*.*71 ± 0*.*04^#^	0.05
Het (n = 33)	78*.*77 ± 0*.*05	0.06	78*.*80 ± 0*.*03	0.04	78*.*69 ± 0*.*04	0.06	78*.*75 ± 0*.*06	0.07

Melting temperatures (Tm) from pcPCR-HRM analysis (mean ± SD), with their corresponding coefficients of variation (CV, %). Tm values of *sapje* samples were significantly higher than Tm values of Het or WT samples in the three runs (*p < 0.0001). Differences in Tm values between WT and Het samples were significant in the first and second run (^#^p = 0.026, ^##^p = 0.0247, and ^###^p = 0.0079, one-way ANOVA with Tukey's multiple comparisons test). A complete dataset is found online in Supplementary Table S2.

**Figure 5:**
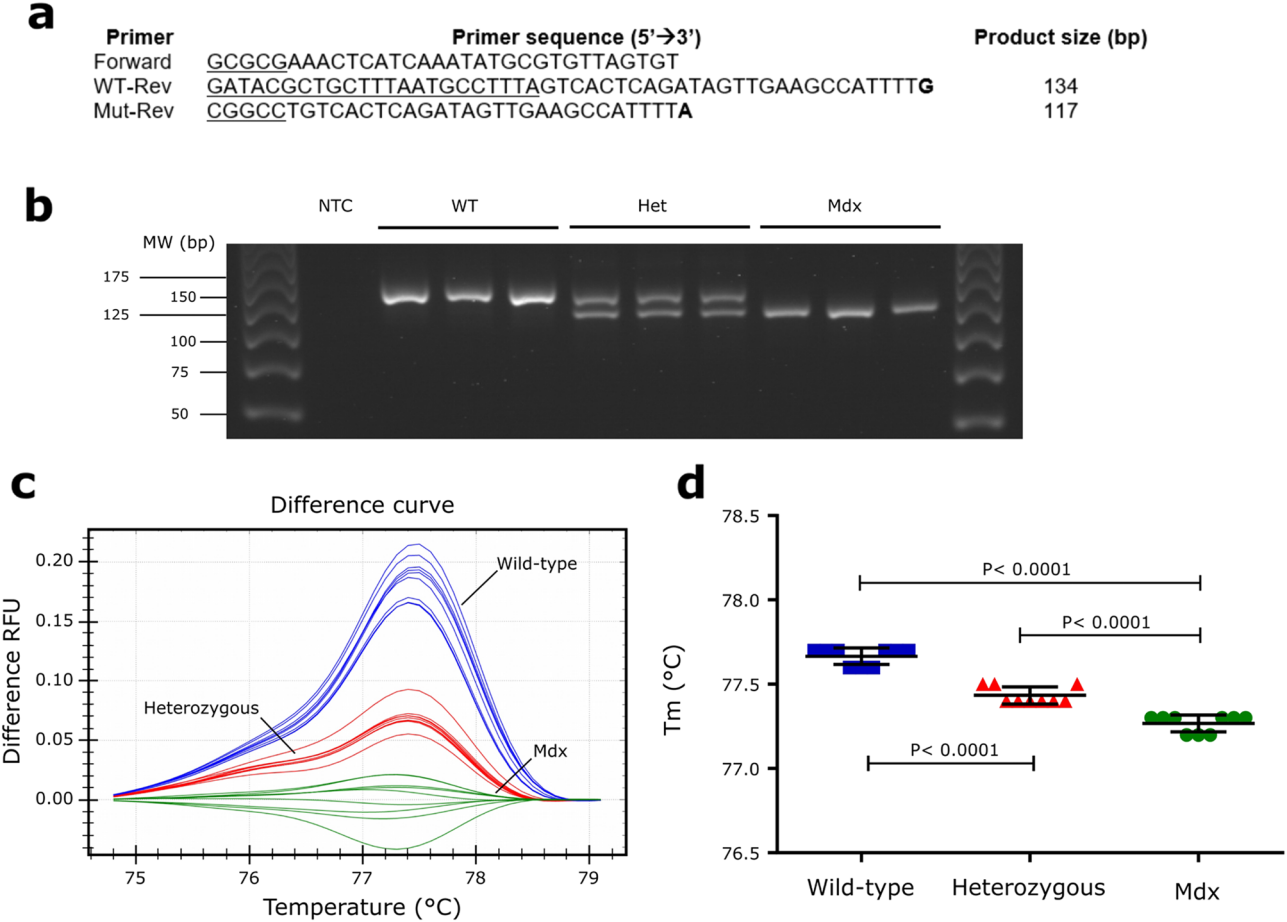
Validation of the pcPCR-HRM technique to genotype mdx mouse. (**a**) Primer sequences and expected product sizes from Shin et al. ^16^. Bold letters represent allele-specific nucleotides at the position of the mdx mutation. Non-specific synthetic tails are underlined. (**b**) Genotypes of three wild-type, three heterozygous and three mdx mice resolved by 3% agarose electrophoresis after pcPCR. (**c**) HRM difference melt plots from 9 mice (same as in **b**) performed in triplicate with the mdx cluster as a baseline. Wild-type (blue), heterozygous (red), and mdx (green) clusters are identified on the melt plot. (**d**) Tm values from pcPCR products (same as in **c**): wild-type (blue), heterozygous (red) and, mdx (green) genotypes. Data are expressed as mean ± SD. One-way ANOVA with Tukey’s multiple comparisons test was used for statistical analysis.

We next wanted to validate our method in another model with a different point mutation. To do this, we used the mdx mouse model of DMD, which carries a nonsense C/T mutation in exon 23. For this study, 9 mouse tails were used, from WT (n = 3), Het (n = 3) and mdx mice (n = 3). We used a primer trio specifically designed for pcPCR, described in a previous study^16^ (Fig. 5a), and we applied the same pcPCR protocol as the one optimized for zebrafish samples. We found that using a hot-start Taq and a 65 °C annealing/extension temperature resulted in robust amplification of DNA with expected sizes with no primer dimers or unspecific bands (Fig. 5b). HRM assay was able to efficiently discriminate WT, Het, and mdx samples in an unbiased manner, based on pcPCR product melt curve profiles (Fig. 5c). After HRM analysis, 100% of samples were automatically assigned to one of the three clusters corresponding to WT (blue), mdx (green), or Het genotypes (red). The melting temperature (Tm) of the pcPCR products from mdx samples (77.27 ± 0.06) were significantly lower compared to WT (77.67 ± 0.06) and Het samples (77.43 ± 0.06; p < 0.0001 for all comparisons), with a decrease in the mean Tm of 0.40 °C and 0.17 °C compared to WT and Het samples, respectively (Fig. 5d). We also observed statistically significant differences in the Tm between WT and Het samples (0.23 °C, p < 0.0001). This result is in agreement with the Tm values calculated by Oligo Calc in silico calculator, which is 0.3 °C lower in mdx PCR products (75.2 °C in WT vs. 74.9 °C in mdx). Overall, these results indicate that our method can be applied to a range of single-nucleotide substitution mutations in different species.

## Discussion

*Sapje* zebrafish is becoming a preferred model for screening novel therapeutic candidates for DMD, and it is also used to study the pathogenic mechanisms of this disease^4,7,8^. Sanger sequencing is the classical genotyping method for *sapje* zebrafish, which carry an A/T point mutation, but this technique may prove costly and time-consuming. Here, we describe a highly reproducible PCR protocol that uses a primer trio for *sapje* genotyping. This method can be easily implemented in a routine laboratory setting, since it may be resolved by standard agarose electrophoresis. Moreover, it can also be used in combination with HRM analysis, which enables unbiased identification of a large number of samples. Indeed, in a single PCR-HRM setup, 128 larvae can be genotyped in triplicate in a 384-well plate with minimal hands-on time. We estimate that these samples could be processed for DNA extraction and analysed by HRM in less than 4.5 h. Given that a large number of samples is relatively common when working with zebrafish, we propose the pcPCR followed by HRM analysis as the most convenient method for genotyping the *sapje* DMD model. Compared to agarose electrophoresis, HRM is less time-consuming, and an automatic cluster assignment removes any potential bias in the interpretation of results.

In the current pcPCR-HRM assay, both the melting curve profiles and Tm values can be used to discriminate *sapje* samples. The accuracy of the melt curve analysis was 97% (2 unresolved samples), with a 94% sensitivity and 98% specificity. Subsequent agarose electrophoresis analysis was able to identify the unresolved samples, reaching 100% accuracy. The intra- and inter-assay variability analysis of Tm values rendered minimum CV values, which indicates an excellent precision and repeatability of the pcPCR-HRM method.

Our genotyping approach presents several advantages over currently used methods to genotype animal models. Compared to conventional pcPCR, our method has the capacity of high specificity and sensitivity. It is also more versatile, faster and unbiased, while allowing easy transition to high-throughput scale. Compared to standard HRM, our method is able to detect challenging point mutations, such as A/T substitutions. It is also more robust and versatile, since genotypes can be resolved through different analysis, alone or in combination, i.e., Tm analysis, HRM difference plots, and/or agarose electrophoresis. Most interestingly, our method is compatible with poor-quality DNA, while standard HRM is susceptible to variations in DNA quality, leading to unreliable genotyping^17^.

In summary, the genotyping method described in this study provides a precise and straightforward alternative to differentiate between WT, Het and mutant genotypes. Subsequent genotype identification may be performed by HRM assay, agarose gel electrophoresis, or even Tm analysis, which makes this method extremely versatile, user-friendly, and accessible at minimum cost and hands-on time. HRM analysis may be used in combination with pcPCR to genotype A/T and C/T point mutations present in the zebrafish and mouse models of DMD^3,16^. Furthermore, this approach is applicable to detect a variety of point mutations and even small insertions or deletions in different species. Implementation of this technique would require a careful primer design for specific PCR amplification, with non-specific tails to generate different-sized products. Using a hot-start Taq polymerase and optimizing the annealing temperature are critical measures for obtaining specific PCR products. More sophisticated PCR cycling protocols may be implemented if needed, such as touch-down or touch-up gradient amplification protocols^18^. Given its robust performance with crude DNA extracts, our approach may be particularly well-suited for detecting single nucleotide variants in poor-quality samples such as ancient DNA or DNA from formalin-fixed, paraffin-embedded material.

## Methods

### Animals

The dmd/sapta222a mutant strain (*sapje*) was obtained from the Tübingen Stock Collection (Tübingen, Germany^19^). Sapta222a heterozygotes were raised and maintained in the zebrafish facility at Biobide following the European Directive (2010/63/EU) for the protection of animals used for scientific purposes and standard procedures, as previously described^20^. C57BL/10ScSn-DMDmdx/J mice (mdx) and C57BL/10ScSnJ mice (wild-type), were obtained from The Jackson Laboratory (Bar Harbor, ME, USA). All experiments were approved by the Ethical Committee for Animal Experimentation at Biodonostia. Crosses of Sapta222a heterozygotes results in WT (25%), Het (50%), and homozygous Sapta222a larvae (*sapje*, 25%). Larvae used for these experiments were 6 dpf or younger.

### Birefringence analysis

Muscle damage in 3–4 dpf *sapje* larvae can be readily detected through a disrupted birefringence pattern^5^. Birefringence is the bright light pattern produced by the diffraction of polarized light through the muscle sarcomeres. For birefringence analysis, larvae were anesthetized with 0.02% tricaine, carefully aligned between two glass-polarizing filters, and viewed with a stereomicroscope while one filter was rotated to maximize birefringence. Larvae consistently showing bright, well-organized somites were phenotyped as unaffected, while those displaying patchy areas of disrupted and disorganized somites were phenotyped as *sapje*.

### DNA extraction

Crude DNA was extracted from whole zebrafish larvae or mouse tails using fast alkaline lysis protocols^11,21^. Briefly, zebrafish larvae were lysed in 20 µl of 50 mM NaOH and heated at 95 °C for 10 min. Mouse tails (~ 2 mm) were lysed in 75 µl of 25 mM NaOH, 0.2 mM EDTA (pH 12) and heated at 100 °C for 30 min. Samples were cooled to 4 °C and neutralized with 2 µl of 1 M Tris–HCl at pH 8.0 (larvae), or 75 µl of 40 mM Tris–HCl at pH 5.0 (mouse tails).

### Primers design

A primer pair was designed for High-Resolution Melt (HRM) and Sanger analyses, with the *sapje* point mutation located in the middle of the PCR product. For the primer competition PCR (pcPCR), a trio of primers was designed consisting of a common forward primer (pcPCR-For), a mutant allele-specific reverse primer (pcPCR-Mut-Rev), and a wild-type allele-specific reverse primer (pcPCR-WT-Rev). Specific primers were designed using Primer Express software (Thermo Fisher), with a 58-60 °C Tm criteria. Then, short GC-rich sequences were added to pcPCR-For and pcPCR-Mut-Rev primers, and a non-specific synthetic tail was added to the pcPCR-WT-Rev primer to allow size discrimination of WT and mutant alleles, based on a previous study^16^. Table 1 shows the sequences of all custom primers used in this study.

### Standard PCR for HRM assay and Sanger analysis

Standard PCRs were carried out with Precision Melt Supermix (Bio-Rad), 300 nM of each primer (HRM-For and HRM-Rev, Table 1) and 1 ng/µl of DNA sample. PCR was run according to the following conditions: an initial denaturation step at 95 °C for 3 min followed by 39 cycles of 95 °C for 10 s and 60 °C for 1 min. HRM analysis was performed in triplicate on a CFX384 Touch PCR equipment (Bio-Rad), over a temperature gradient of 65–95 °C with a 0.1 °C increment, pausing for 5 s before each increment. Genotypes were resolved by examining normalized and difference melt plots using the Precision Melt Analysis software (Bio-Rad). Sanger sequencing was carried out by the Genomic Platform of Biodonostia Institute, using the HRM-Rev primer and a 16-capillary ABI 3130xl platform (Applied Biosystems) according to the manufacturer’s protocol. Some samples could not be resolved by Sanger sequencing, likely due to the crude DNA extraction method.

### Primer competition PCR

Primer competition PCR (pcPCR) was carried out with Precision Melt Supermix (Bio-Rad) with a hot-start Taq DNA polymerase (iTaq), 200 nM of each custom-designed primer (one forward and two reverse primers), and 5% of DNA solution. The PCR protocol comprised an initial denaturation step at 95 °C for 3 min, followed by 34 cycles of 95 °C for 10 s and 65 °C for 1 min. Primer sequences and expected PCR products for WT and mutant samples are detailed in Table 1 for the zebrafish model, and in Fig. 5 for the mouse model. HRM analysis was performed as described above, and PCR products were subsequently resolved by 3% agarose gel electrophoresis. Repeatability of the pcPCR-HRM analysis in zebrafish samples was assessed by determination of the intra- and inter-assay CV of the Tm of three runs carried out on different days.

### Statistical analysis

Data distribution was evaluated with D'Agostino & Pearson omnibus normality test (GraphPad Prism 6). Statistical significance was determined using One-Way ANOVA followed by unpaired Tukey’s posthoc test. The adjusted *P* values of less than 0.05 were considered statistically significant.

## Supplementary information

Supplementary Information

## Data Availability

Data generated during the study is presented in an analysed format in this manuscript. Raw datasets generated from the intra- and inter-assays are included in the Supplementary Information file.
