# Supplementary material for: A genotyping method combining primer competition PCR with HRM analysis to identify point mutations in Duchenne animal models
Source: Sci Rep. 2020 Oct 14;10:17224. doi: 10.1038/s41598-020-74173-y (PMC7560699; doi:10.1038/s41598-020-74173-y)
Supplement: Supplementary file 1 — Supplementary Information [file 41598_2020_74173_MOESM1_ESM.pdf]

A genotyping method combining primer competition PCR with HRM analysis to identify point mutations in Duchenne animal models

Haizpea Lasa-Fernandez, Laura Mosqueira-Martín, Ainhoa Alzualde, Jaione Lasa-Elgarresta, and Ainara Vallejo-Illarramendi

Supplementary information

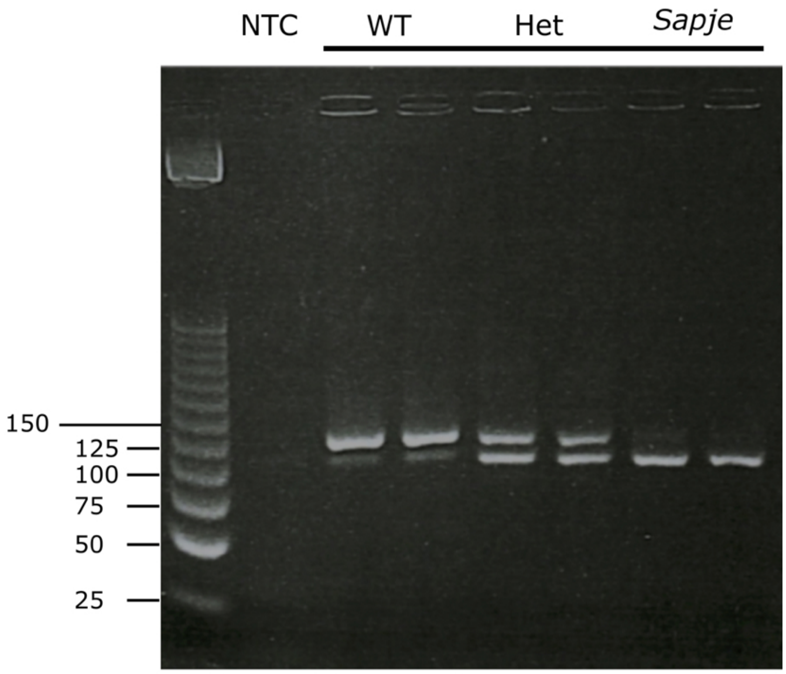

Supplementary Figure S1. Optimization of the pcPCR extension and annealing temperature. pcPCR amplification at 60ºC results in non-specific additional bands in homozygous samples. These unspecific bands are not observed or greatly diminished at 65ºC (Figures 2, 3).

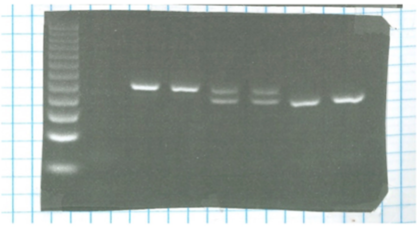

Figure 2

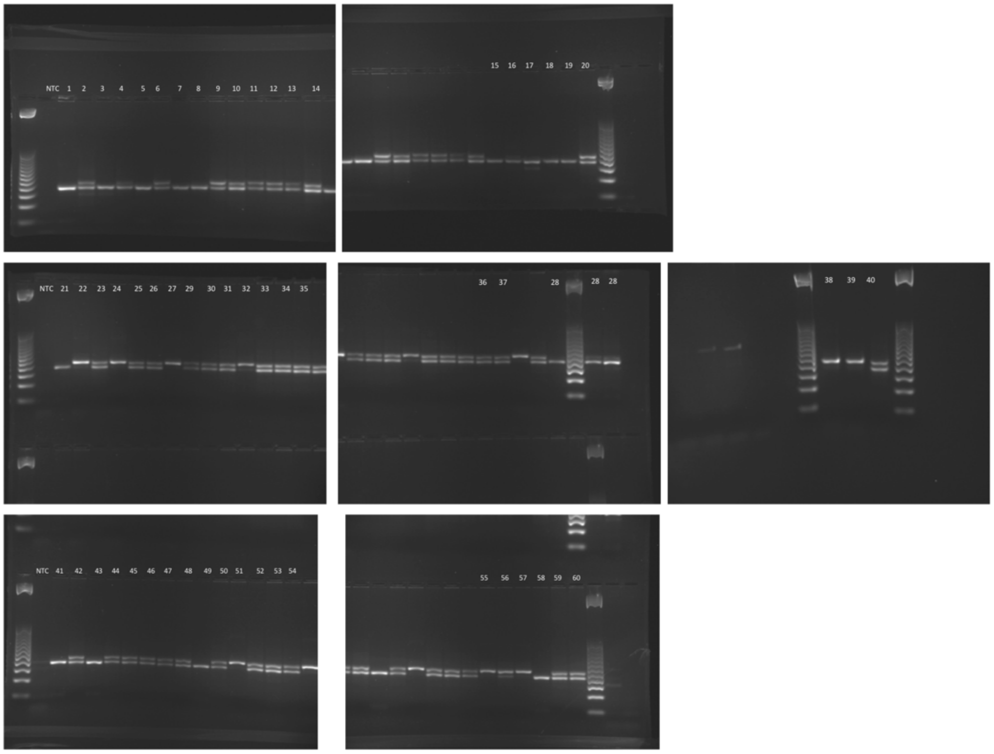

Figure 3

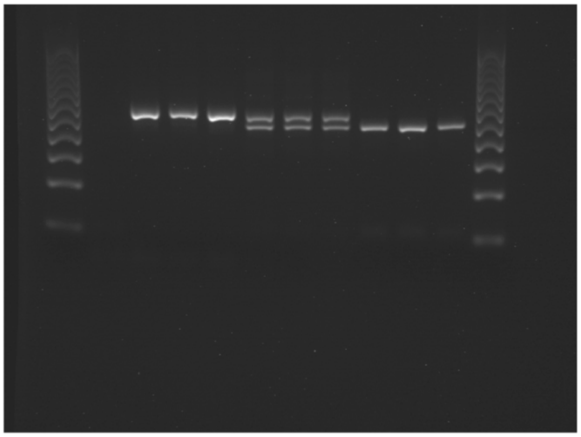

Figure 5 blots

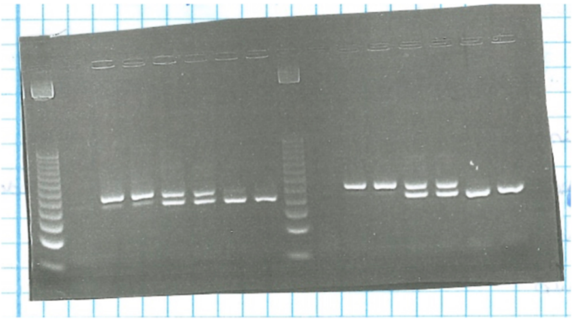

Supplementary Figure 1

Supplementary Figure S2. Uncropped original images from agarose gels

Supplementary Table S1. Genotype and phenotype analyses in 60 zebrafish larvae. *Sapje* phenotype was determined by birefringence analysis. HRM data shows genotypes resolved by HRM analysis of pcPCR products. Cluster, percentage confidence (highest % value), and melting temperature (Tm, mean ± SD) values are extracted from the HRM analysis. Gel refers to genotypes resolved by 3% agarose gel electrophoresis. Seq refers to genotypes resolved by Sanger sequencing.

Sample	Phenotype	Cluster	Confidence %	Tm	HRM	Gel	Seq
1	*Sapje*	2	99.8	79.17 ± 0.06	*Sapje*	*Sapje*	*Sapje*
2	Unaffected	1	98.0	78.77 ± 0.06	Het	Het	
3	*Sapje*	2	98.7	79.13 ± 0.06	*Sapje*	*Sapje*	
4	*Sapje*	2	92.9	78.9 ± 0.0	*Sapje*	*Sapje*	
5	*Sapje*	2	99.9	79.1 ± 0.0	*Sapje*	*Sapje*	
6	Unaffected	1	99.4	78.8 ± 0.0	Het	Het	Het
7	*Sapje*	2	94.2	78.97 ± 0.06	*Sapje*	*Sapje*	
8	*Sapje*	2	99.3	79.13 ± 0.06	*Sapje*	*Sapje*	*Sapje*
9	Unaffected	1	94.6	78.77 ± 0.06	Het	Het	Het
10	Unaffected	1	98.1	78.83 ± 0.06	Het	Het	
11	Unaffected	1	99.3	78.8 ± 0.0	Het	Het	
12	Unaffected	1	99.4	78.77 ± 0.06	Het	Het	
13	Unaffected	1	99.7	78.8 ± 0.0	Het	Het	
14	Unaffected	1	98.5	78.77 ± 0.06	Het	Het	
15	*Sapje*	2	99.5	79.1 ± 0.0	*Sapje*	*Sapje*	
16	*Sapje*	2	97.9	79 ± 0.10	*Sapje*	*Sapje*	*Sapje*
17	*Sapje*	2	98.0	79.1 ± 0.0	*Sapje*	*Sapje*	
18	*Sapje*	2	99.6	79.1 ± 0.0	*Sapje*	*Sapje*	
19	*Sapje*	2	99.6	79.1 ± 0.0	*Sapje*	*Sapje*	
20	Unaffected	1	94.5	78.7 ± 0.0	Het	Het	
21	*Sapje*	2	99.3	79.1 ± 0.0	*Sapje*	*Sapje*	*Sapje*
22	Unaffected	3	98.4	78.7 ± 0.0	WT	WT	WT
23	Unaffected	1	99.6	78.8 ± 0.0	Het	Het	Het
24	Unaffected	3	98.6	78.7 ± 0.0	WT	WT	
25	Unaffected	1	99.8	78.7 ± 0.17	Het	Het	
26	Unaffected	1	99.5	78.8 ± 0.0	Het	Het	
27	Unaffected	3	98.3	78.7 ± 0.0	WT	WT	WT
28	*Sapje*	2	60.4	78.9	*Sapje*	*Sapje*	*Sapje*
		1	97.7	78.9	Het		
		2	98.0	79	*Sapje*		
29	Unaffected	1	99.4	78.8 ± 0.0	Het	Het	
30	Unaffected	1	99.8	78.8 ± 0.0	Het	Het	
31	Unaffected	1	99.4	78.8 ± 0.0	Het	Het	
32	Unaffected	3	98.7	78.7 ± 0.0	WT	WT	
33	Unaffected	1	99.2	78.77 ± 0.06	Het	Het	
34	Unaffected	1	99.7	78.8 ± 0.0	Het	Het	
35	Unaffected	1	99.5	78.8 ± 0.0	Het	Het	
36	Unaffected	1	99.6	78.7 ± 0.0	Het	Het	
37	Unaffected	1	99.3	78.8 ± 0.0	Het	Het	
38	Unaffected	3	98.2	78.7 ± 0.0	WT	WT	
39	Unaffected	3	98.2	78.7 ± 0.0	WT	WT	WT

40	Unaffected	1	71.7	78.6	Het	Het	Het
		1	98.3	78.7	Het		
		3	92.6	78.6	WT		
41	*Sapje*	2	99.3	79.1 ± 0.0	*Sapje*	*Sapje*	*Sapje*
42	Unaffected	1	99.5	78.8 ± 0.0	Het	Het	
43	*Sapje*	2	99.6	79 ± 0.0	*Sapje*	*Sapje*	*Sapje*
44	Unaffected	1	99.7	78.7 ± 0.0	Het	Het	
45	Unaffected	1	99.6	78.8 ± 0.0	Het	Het	
46	Unaffected	1	99.7	78.7 ± 0.0	Het	Het	Het
47	Unaffected	1	99.5	78.77 ± 0.06	Het	Het	
48	Unaffected	1	99.1	78.7 ± 0.0	Het	Het	
49	*Sapje*	2	99.6	79.1 ± 0.0	*Sapje*	*Sapje*	
50	Unaffected	1	99.7	78.8 ± 0.0	Het	Het	
51	Unaffected	3	98.7	78.6 ± 0.17	WT	WT	
52	Unaffected	1	99.4	78.77 ± 0.06	Het	Het	
53	Unaffected	1	99.8	78.73 ± 0.06	Het	Het	
54	Unaffected	1	99.7	78.73 ± 0.06	Het	Het	Het
55	Unaffected	3	99.2	78.77 ± 0.06	WT	WT	WT
56	Unaffected	3	99.2	78.73 ± 0.06	WT	WT	WT
57	Unaffected	3	98.7	78.73 ± 0.06	WT	WT	
58	*Sapje*	2	98.9	79.1 ± 0.0	*Sapje*	*Sapje*	
59	Unaffected	1	99.6	78.77 ± 0.06	Het	Het	
60	Unaffected	1	99.7	78.8 ± 0.0	Het	Het	

Supplementary Table S2. Raw dataset and statistical parameters generated from the intra- and inter-assays using 60 zebrafish larvae. Melting temperatures (Tm, Mean, and SD) from 3 pcPCR-HRM independent runs, with their corresponding coefficients of variation (CV, %).

		Run 1					Run 2					Run 3					Inter-assay			
Genotype	Sample	Tm	Mean	SD	CV %	Intra-assay	Tm	Mean	SD	CV %	Intra-assay	Tm	Mean	SD	CV %	Intra-assay	Mean	SD	CV %	Inter-assay
			Tm			CV%		Tm			CV%		Tm			CV%	Tm			CV%
	1	79.1	79.17	0.06	0.07		79.2	79.2	0	0		79.1	79.1	0	0		79.16	0.05	0.06	
		79.2					79.2					79.1								
		79.2					79.2					79.1								
	3	79.2	79.13	0.06	0.07		79.2	79.13	0.06	0.07		79.1	79.1	0	0		79.12	0.02	0.02	
		79.1					79.1					79.1								
		79.1					79.1					79.1								
	4	78.9	78.9	0	0		79.0	79.0	0	0		79.1	79.1	0	0		79	0.10	0.13	
		78.9					79.0					79.1								
		78.9					79.0					79.1								
	5	79.1	79.1	0	0		79.2	79.13	0.06	0.07		79.1	79.07	0.06	0.07		79.1	0.03	0.04	
		79.1					79.1					79.1								
		79.1					79.1					79.0								
	7	79.0	78.97	0.06	0.07		79.0	78.97	0.06	0.07		78.8	78.8	0	0		78.91	0.10	0.12	
		79.0					79.0					78.8								
		78.9					78.9					78.8								
	8	79.2	79.13	0.06	0.07		79.2	79.17	0.06	0.07		79.1	79.1	0	0		79.13	0.03	0.04	
		79.1					79.2					79.1								
		79.1					79.1					79.1								
	15	79.1	79.1	0	0		79.1	79.1	0	0		79.1	79.1	0	0		79.1	0	0	
		79.1					79.1					79.1								
		79.1					79.1					79.1								
	16	78.9	79	0.10	0.13		79.0	79.07	0.06	0.07		79.2	79.13	0.06	0.07		79.07	0.07	0.08	
		79.0					79.1					79.1								
		79.1					79.1					79.1								
	17	79.1	79.1	0	0		79.1	79.1	0	0		79.1	79.1	0	0		79.1	0	0	
Sapje		79.1					79.1					79.1								
		79.1					79.1					79.1								
	18	79.1	79.1	0	0		79.1	79.13	0.06	0.07		79.1	79.1	0	0		79.11	0.02	0.02	
		79.1					79.1					79.1								
		79.1					79.2					79.1								
	19	79.1	79.1	0	0		79.1	79.17	0.06	0.07		79.1	79.1	0	0		79.12	0.04	0.05	
		79.1					79.2					79.1								
		79.1					79.2					79.1								
	21	79.1	79.1	0	0		79.2	79.13	0.06	0.07		79.0	79,00	0	0		79.08	0.07	0.09	
		79.1					79.1					79.0								
		79.1					79.1					79.0								
	28	78.9	78.93	0.06	0.07		79.0	79.03	0.06	0.07		79.1	79.1	0	0		79.02	0.08	0.11	
		78.9					79.0					79.1								
		79.0					79.1					79.1								
	41	79.1	79.1	0	0		79.1	79.1	0	0		79.1	79.07	0.06	0.07		79.09	0.02	0.02	
		79.1					79.1					79.1								
		79.1					79.1					79.0								
	43	79.0	79	0	0		79.1	79.07	0.06	0.07		78.8	78.8	0	0		78.96	0.14	0.18	
		79.0					79.1					78.8								
		79.0					79.0					78.8								
	49	79.1	79.1	0	0		79.1	79.1	0	0		79.0	79.03	0.06	0.07		79.08	0.04	0.05	
		79.1					79.1					79.1								
		79.1					79.1					79.0								
	58	79.1	79.1	0	0		79.1	79.1	0	0		79.0	79	0	0		79.07	0.06	0.07	
		79.1					79.1					79.0								
		79.1					79.1					79.0								
	**Total**		**79.07**	**0.08**		**0.10**		**79.10**	**0.06**		**0.08**		**79.05**	**0.10**		**0.13**	**79.07**	**0.03**		**0.03**

**WT**	22	78.7<br>78.7<br>78.7	78.7	0	0		78.8<br>78.8<br>78.7	78.77	0.06	0.07		78.7<br>78.7<br>78.7	78.7	0	0		78.72	0.04	0.05	
	24	78.7<br>78.7<br>78.7	78.7	0	0		78.7<br>78.7<br>78.7	78.7	0	0		78.7<br>78.7<br>78.7	78.7	0	0		78.7	0	0	
	27	78.7<br>78.7<br>78.7	78.7	0	0		78.8<br>78.8<br>78.7	78.77	0.06	0.07		78.7<br>78.7<br>78.7	78.7	0	0		78.72	0.04	0.05	
	32	78.7<br>78.7<br>78.7	78.7	0	0		78.8<br>78.8<br>78.8	78.8	0	0		78.8<br>78.7<br>78.8	78.77	0.06	0.07		78.76	0.05	0.06	
	38	78.7<br>78.7<br>78.7	78.7	0	0		78.8<br>78.7<br>78.7	78.73	0.06	0.07		78.6<br>78.6<br>78.6	78.6	0	0		78.68	0.07	0.09	
	39	78.7<br>78.7<br>78.7	78.7	0	0		78.8<br>78.7<br>78.7	78.73	0.06	0.07		78.7<br>78.7<br>78.7	78.7	0	0		78.71	0.02	0.02	
	51	78.7<br>78.7<br>78.4	78.6	0.17	0.22		78.7<br>78.7<br>78.5	78.63	0.12	0.15		78.6<br>78.6<br>78.6	78.6	0	0		78.61	0.02	0.02	
	55	78.7<br>78.8<br>78.8	78.77	0.06	0.07		78.8<br>78.8<br>78.8	78.8	0	0		78.6<br>78.6<br>78.6	78.6	0	0		78.72	0.11	0.14	
	56	78.7<br>78.7<br>78.8	78.73	0.06	0.07		78.8<br>78.8<br>78.8	78.8	0	0		78.7<br>78.6<br>78.6	78.63	0.06	0.07		78.72	0.08	0.11	
	57	78.7<br>78.7<br>78.8	78.73	0.06	0.07		78.8<br>78.8<br>78.8	78.8	0	0		78.7<br>78.7<br>78.7	78.7	0	0		78.74	0.05	0.06	
	**Total**		**78.70**	**0.04**		**0.05**		**78.75**	**0.05**		**0.07**		**78.67**	**0.06**		**0.07**	**78.71**	**0.04**		**0.05**
**Het**	2	78.8<br>78.8<br>78.7	78.77	0.06	0.07		78.8<br>78.8<br>78.8	78.8	0	0		78.6<br>78.6<br>78.6	78.8	0	0		78.72	0.11	0.14	
	6	78.8<br>78.8<br>78.8	78.8	0	0		78.8<br>78.8<br>78.8	78.8	0	0		78.7<br>78.7<br>78.7	78.7	0	0		78.77	0.06	0.07	
	9	78.8<br>78.7<br>78.8	78.77	0.06	0.07		78.8<br>78.8<br>78.8	78.8	0	0		78.7<br>78.7<br>78.7	78.7	0	0		78.76	0.05	0.06	
	10	78.9<br>78.8<br>78.8	78.83	0.06	0.07		78.9<br>78.9<br>78.8	78.87	0.06	0.07		78.6<br>78.6<br>78.6	78.6	0	0		78.77	0.15	0.18	
	11	78.8<br>78.8<br>78.8	78.8	0	0		78.8<br>78.8<br>78.8	78.8	0	0		78.7<br>78.7<br>78.7	78.7	0	0		78.77	0.06	0.07	
	12	78.8<br>78.7<br>78.8	78.77	0.06	0.07		78.8<br>78.8<br>78.8	78.8	0	0		78.7<br>78.7<br>78.7	78.7	0	0		78.76	0.05	0.06	
	13	78.8<br>78.8<br>78.8	78.8	0	0		78.8<br>78.8<br>78.8	78.8	0	0		78.7<br>78.7<br>78.7	78.7	0	0		78.77	0.06	0.07	
	14	78.7<br>78.8<br>78.8	78.77	0.06	0.07		78.8<br>78.8<br>78.8	78.8	0	0		78.8<br>78.8<br>78.7	78.77	0.06	0.07		78.78	0.02	0.02	
	20	78.7<br>78.7<br>78.7	78.7	0	0		78.8<br>78.8<br>78.8	78.8	0	0		78.7<br>78.7<br>78.7	78.7	0	0		78.73	0.06	0.07	
	23	78.8<br>78.8<br>78.8	78.8	0	0		78.8<br>78.8<br>78.8	78.8	0	0		78.7<br>78.7<br>78.7	78.7	0	0		78.77	0.06	0.07	
	25	78.8<br>78.8<br>78.5	78.7	0.17	0.22		78.8<br>78.8<br>78.6	78.73	0.12	0.15		78.8<br>78.8<br>78.7	78.77	0.06	0.07		78.73	0.03	0.04	
	26	78.8<br>78.8<br>78.8	78.8	0	0		78.8<br>78.8<br>78.8	78.8	0	0		78.8<br>78.7<br>78.7	78.73	0.06	0.07		78.78	0.04	0.05	
	29	78.8<br>78.8	78.8	0	0		78.8<br>78.8	78.8	0	0		78.7<br>78.7	78.7	0	0		78.77	0.06	0.07	

		78.8					78.8					78.7								
	30	78.8<br>78.8<br>78.8	78.8	0	0		78.8<br>78.8<br>78.8	78.8	0	0		78.7<br>78.7<br>78.7	78.7	0	0		78.77	0.06	0.07	
	31	78.8<br>78.8<br>78.8	78.8	0	0		78.8<br>78.8<br>78.8	78.8	0	0		78.7<br>78.7<br>78.7	78.7	0	0		78.77	0.06	0.07	
	33	78.8<br>78.8<br>78.7	78.77	0.06	0.07		78.9<br>78.9<br>78.8	78.87	0.06	0.07		78.7<br>78.7<br>78.7	78.7	0	0		78.78	0.08	0.11	
	34	78.8<br>78.8<br>78.8	78.8	0	0		78.8<br>78.8<br>78.8	78.8	0	0		78.7<br>78.7<br>78.7	78.7	0	0		78.77	0.06	0.07	
	35	78.8<br>78.8<br>78.8	78.8	0	0		78.8<br>78.8<br>78.8	78.8	0	0		78.7<br>78.7<br>78.7	78.7	0	0		78.77	0.06	0.07	
	36	78.7<br>78.7<br>78.7	78.7	0	0		78.8<br>78.8<br>78.8	78.8	0	0		78.7<br>78.7<br>78.7	78.7	0	0		78.73	0.06	0.07	
	37	78.8<br>78.8<br>78.8	78.8	0	0		78.8<br>78.8<br>78.8	78.8	0	0		78.7<br>78.7<br>78.7	78.7	0	0		78.77	0.06	0.07	
	40	78.6<br>78.7<br>78.6	78.63	0.06	0.07		78.7<br>78.7<br>78.7	78.7	0	0		78.6<br>78.6<br>78.6	78.6	0	0		78.64	0.05	0.06	
	42	78.8<br>78.8<br>78.8	78.8	0	0		78.8<br>78.8<br>78.8	78.8	0	0		78.7<br>78.6<br>78.7	78.67	0.06	0.07		78.76	0.08	0.10	
	44	78.7<br>78.7<br>78.7	78.7	0	0		78.8<br>78.7<br>78.7	78.73	0.06	0.07		78.7<br>78.7<br>78.7	78.7	0	0		78.71	0.02	0.02	
	45	78.8<br>78.8<br>78.8	78.8	0	0		78.8<br>78.8<br>78.8	78.8	0	0		78.7<br>78.7<br>78.7	78.7	0	0		78.77	0.06	0.07	
	46	78.7<br>78.7<br>78.7	78.7	0	0		78.8<br>78.8<br>78.8	78.8	0	0		78.7<br>78.7<br>78.7	78.7	0	0		78.73	0.06	0.07	
	47	78.7<br>78.8<br>78.8	78.77	0.06	0.07		78.8<br>78.8<br>78.8	78.8	0	0		78.6<br>78.6<br>78.6	78.6	0	0		78.72	0.11	0.14	
	48	78.7<br>78.7<br>78.7	78.7	0	0		78.7<br>78.8<br>78.8	78.77	0.06	0.07		78.7<br>78.7<br>78.7	78.7	0	0		78.72	0.04	0.05	
	50	78.8<br>78.8<br>78.8	78.8	0	0		78.8<br>78.8<br>78.8	78.8	0	0		78.7<br>78.7<br>78.7	78.7	0	0		78.77	0.06	0.07	
	52	78.7<br>78.8<br>78.8	78.77	0.06	0.07		78.8<br>78.8<br>78.8	78.8	0	0		78.6<br>78.6<br>78.6	78.6	0	0		78.72	0.11	0.14	
	53	78.8<br>78.7<br>78.7	78.73	0.06	0.07		78.8<br>78.8<br>78.8	78.8	0	0		78.7<br>78.7<br>78.7	78.7	0	0		78.74	0.05	0.06	
	54	78.7<br>78.7<br>78.8	78.73	0.06	0.07		78.8<br>78.8<br>78.8	78.8	0	0		78.7<br>78.7<br>78.7	78.7	0	0		78.74	0.05	0.06	
	59	78.7<br>78.8<br>78.8	78.77	0.06	0.07		78.8<br>78.8<br>78.9	78.83	0.06	0.07		78.7<br>78.7<br>78.7	78.7	0	0		78.77	0.07	0.08	
	60	78.8<br>78.8<br>78.8	78.8	0	0		78.8<br>78.8<br>78.8	78.8	0	0		78.6<br>78.6<br>78.6	78.6	0	0		78.73	0.12	0.15	
	**Total**		**78.77**	**0.05**		**0.06**		**78.80**	**0.03**		**0.04**		**78.69**	**0.04**		**0.06**	**78.75**	**0.06**		**0.07**
